# The Prevalence and Prognosis of Cachexia in Patients with Non-Sarcopenic Dysphagia: A Retrospective Cohort Study

**DOI:** 10.3390/nu16172917

**Published:** 2024-09-01

**Authors:** Shingo Kakehi, Hidetaka Wakabayashi, Takako Nagai, Shinta Nishioka, Eri Isono, Yukiko Otsuka, Junki Ninomiya, Ryo Momosaki

**Affiliations:** 1 Department of Rehabilitation Medicine, Tokyo Women’s Medical University Hospital, Tokyo 162-8666, Japan; kakehi.shingo@twmu.ac.jp (S.K.);; 2 Department of Clinical Nutrition and Food Service, Nagasaki Rehabilitation Hospital, Nagasaki 850-0854, Japan; 3 Department of Nursing, Tokyo Women’s Medical University Hospital, Tokyo 162-8666, Japan; 4 Department of Rehabilitation Medicine, Mie University Graduate School of Medicine, Tsu City 514-8507, Japan

**Keywords:** aged, deglutition disorders, nutrition, rehabilitation, sarcopenia, cachexia

## Abstract

The purpose of this study was to assess the prevalence and prognosis of cachexia in patients with non-sarcopenic dysphagia. A retrospective cohort study was conducted using the Japanese sarcopenic dysphagia database. Cachexia was diagnosed using the Asian Working Group for Cachexia criteria, sarcopenia using the Asian Working Group for Sarcopenia 2019 criteria, and malnutrition using the Global Leadership Initiative on Malnutrition criteria. Outcomes were death, swallowing function (Food Intake LEVEL Scale (FILS)), and activities of daily living (Barthel Index (BI)). The mean age of the 175 non-sarcopenic dysphagia patients was 77 (±11) years; 103 (59%) were male, 30 (17%) had cachexia, 133 (76%) had whole-body sarcopenia, and 92 (53%) were malnourished. Of the 30 patients with cachexia, 4 and 11 did not have sarcopenia and malnutrition, respectively. No significant associations were found between cachexia, sarcopenia, and malnutrition. Death was notably higher in the cachexia group (5/30; 17% vs. 2/145; 1%, *p* = 0.002). Median FILS (7 vs. 8, *p* = 0.585) and median BI (35 vs. 50, *p* = 0.469) scores did not show significant differences based on cachexia status. The prevalence of cachexia was 17%, and mortality may be higher with cachexia in non-sarcopenic dysphagia patients.

## 1. Introduction

Dysphagia is common in older people and is often associated with malnutrition and sarcopenia. Although papers vary, approximately 30% of older people have dysphagia, and its prevalence increases with concomitant cerebrovascular disease and dementia [1,2,3]. Some community-dwelling older adults are at risk of malnutrition and poor swallowing, while hospitalized and institutionalized older adults have an even higher risk of malnutrition due to poor swallowing [4,5,6]. Many patients hospitalized for community-acquired pneumonia have dysphagia, and 75% of patients over the age of 65 with aspiration pneumonia have dysphagia, which increases hospital length of stay and mortality [7,8,9]. Causes of dysphagia include sarcopenia, which involves whole-body weakness or a loss of muscle mass in skeletal and swallowing muscles, and non-sarcopenic causes. Non-sarcopenic causes include neurological diseases (such as stroke, dementia, and Parkinson’s disease), head and neck cancer, etc. Preventing these problems is essential because the combination of dysphagia with malnutrition and sarcopenia is associated with reduced life expectancy, activities of daily living (ADLs), and quality of life (QOL) [10]. However, treatment approaches differ between sarcopenic and non-sarcopenic dysphagia. The treatment and prevention of sarcopenic dysphagia require an improved nutritional status through aggressive nutritional management and enhanced physical function through whole-body and swallowing muscle training [10]. Distinguishing between sarcopenic and non-sarcopenic dysphagia may aid in effective prevention and treatment planning.

Dysphagia may be linked to cachexia, but the prevalence and prognosis of cachexia in patients without sarcopenic dysphagia remain unclear. As stated by the Asian Working Group for Cachexia (AWGC), cachexia is defined as a metabolic disorder linked to chronic diseases characterized by a loss of weight, inflammation, and anorexia [11]. Cancer cachexia significantly increases mortality risk and is linked to decreased physical function, psychological decline, and reduced ADLs and QOL in cancer patients [12,13]. The AWGC criteria are effective in diagnosing cancer cachexia in Asians and provide a better prognostic indicator [14,15]. In fact, 7.8% of diabetic patients [16] and 35.2% of hemodialysis patients [17] meet the AWGC criteria for cachexia, which is associated with the overlap of malnutrition and sarcopenia and with mortality and functional impairment. Although drug therapy and multimodal approaches to cachexia treatment may be helpful [18], multimodal approaches are highly individualized, and further research is needed. The ESMO Clinical Practice Guidelines also indicate that sarcopenia, when it occurs prior to weight loss, is associated with cachexia and malnutrition [18]. Early screening and diagnosis of sarcopenia through the measurement and scoring of limb circumference, which correlates with muscle strength and muscle mass, can facilitate the early diagnosis of cachexia [19,20]. We have previously reported on cachexia in sarcopenic dysphagia [21,22]. Cachexia was found in 36% of patients, and death was significantly more common, regardless of the Food Intake Level Scale (FILS) or the Barthel Index (BI) scores. However, no studies have been conducted on patients with non-sarcopenic dysphagia. We hypothesize that even in patients with non-sarcopenic dysphagia, the prevalence of cachexia would not be low and that it would be associated with death.

The purpose of this study is to examine the prevalence and prognosis of cachexia in patients with non-sarcopenic dysphagia, as stated by the diagnostic criteria of the AWGC and to advocate early diagnosis and intervention for cachexia.

## 2. Materials and Methods

### 2.1. Study Design and Patients

We conducted a retrospective cohort study using the Japanese Sarcopenia Dysphagia Database established by the Rehabilitation Nutrition Database Committee of the Japanese Society of Rehabilitation Nutrition and the Japanese Sarcopenia Dysphagia Working Group. The database has been described in detail in a previous report [23]. Participants were patients with dysphagia aged 20 years or older, with data collected between November 2019 and March 2021. All staff at participating facilities used a data entry manual to ensure data accuracy. The study included patients from various settings, such as acute care hospitals, rehabilitation hospitals, long-term care hospitals, home rehabilitation centers, and other facilities. The inclusion criteria were patients over 20 years of age without sarcopenic dysphagia. The exclusion criteria included a lack of data on sarcopenia definitions, such as muscle mass, muscle strength, and physical function, and the presence of sarcopenic dysphagia. We collected follow-up data at discharge for inpatients and at three months for homebound patients and patients who were admitted more than three months after the baseline assessment. However, only weight loss rate data were used after 6 months. The rationale for collecting follow-up data at three months is that the average stay in a convalescent rehabilitation ward in Japan is 75.9 days [24].

### 2.2. Diagnosis of Sarcopenic Dysphagia

Sarcopenic dysphagia is defined as difficulty swallowing resulting from decreased muscle mass and weakness in the whole body and the muscles involved explicitly in swallowing. Patients without whole-body sarcopenia or those with an identifiable cause of dysphagia, such as a stroke, are excluded from this classification, as their dysphagia is likely attributable to the underlying disease [25]. Therefore, patients with whole-body sarcopenia whose dysphagia is due to an underlying disease are diagnosed with non-sarcopenic dysphagia.

We used a reliable and validated algorithm for the diagnosis of sarcopenic dysphagia [26]. Patients were diagnosed with non-sarcopenic dysphagia if there was no whole-body sarcopenia according to the Asian Working Group for Sarcopenia (AWGS) 2019 criteria [27] or if there was a clear cause of dysphagia. Swallowing muscle strength was assessed by tongue pressure, with a tongue pressure of less than 20 kpa used as the cutoff value for low swallowing muscle strength [27,28]. In this study, we included individuals who did not meet the criteria for sarcopenic dysphagia according to the diagnostic flowchart.

### 2.3. Cachexia Diagnostic Criteria

The AWGC criteria for cachexia were used to identify cachexia [11]. These criteria necessitate the existence of an underlying condition such as cancer, chronic heart failure, chronic obstructive pulmonary disease, chronic kidney disease, rheumatoid arthritis, chronic respiratory failure, and progressive or uncontrolled chronic infections, along with either weight loss exceeding 2% over a period of 3 to 6 months or a low body mass index (BMI) of less than 21 kg/m^2^. Additionally, one or more of the following diagnostic criteria for cachexia must be met:

1. Subjective symptom: loss of appetite;
2. Objective measure: reduced grip strength (less than 28 kg in men and less than 18 kg in women);
3. Biomarker: increased C-reactive protein (CRP) levels (greater than 0.5 mg/dL).

In this study, we divided patients into two groups based on the presence or absence of cachexia.

### 2.4. Outcomes and Other Data

The outcomes measured were death, swallowing function evaluated by the FILS [29], and ADLs evaluated by the BI [30]. Other data included age, sex, setting, main disease, comorbidity related to cachexia, sarcopenia according to the AWGS 2019 criteria, malnutrition according to the Global Leadership Initiative on Malnutrition criteria [31], BMI, body weight loss over 6 months, handgrip strength, CRP, and causes of death.

FILS levels 1–3 represent different stages of non-oral feeding, levels 4–6 reflect various combinations of oral and alternative feeding, levels 7–8 correspond to degrees of oral feeding only, level 9 signifies no dietary restrictions with some medical considerations, and level 10 denotes normal oral feeding.

The BI comprises 10 items, each evaluated as ‘full assistance’, ‘partial assistance’, or ‘independent’, with a maximum possible score of 100 points. Walking and transferring between a wheelchair and bed are scored at 0, 5, 10, or 15 points. Activities such as feeding, toileting, stair navigation, dressing, bowel control, and bladder control are scored at 0, 5, or 10 points. Grooming and bathing are scored at 0 or 5 points. A higher total score reflects a greater level of independence.

### 2.5. Statistical Analysis

Statistical analyses were performed using the IBM Statistical Package for the Social Sciences (SPSS) version 26 software (IBM Corporation, Armonk, NY, USA). The normality of the data was checked with the Shapiro–Wilk and Kolmogorov–Smirnov tests. Parametric data were presented as means ± standard deviation (SD), whereas nonparametric data were reported as medians with interquartile ranges (IQRs). The Chi-squared test, Mann–Whitney U test, and *t*-test were employed to analyze baseline and follow-up characteristics, and differences between patients with and without cachexia. Specifically, the Mann–Whitney U test was applied to non-normally distributed quantitative variables, while the Chi-squared test was used for categorical variables. A *p* value of less than 0.05 was considered statistically significant.

## 3. Results

**Figure 1:**
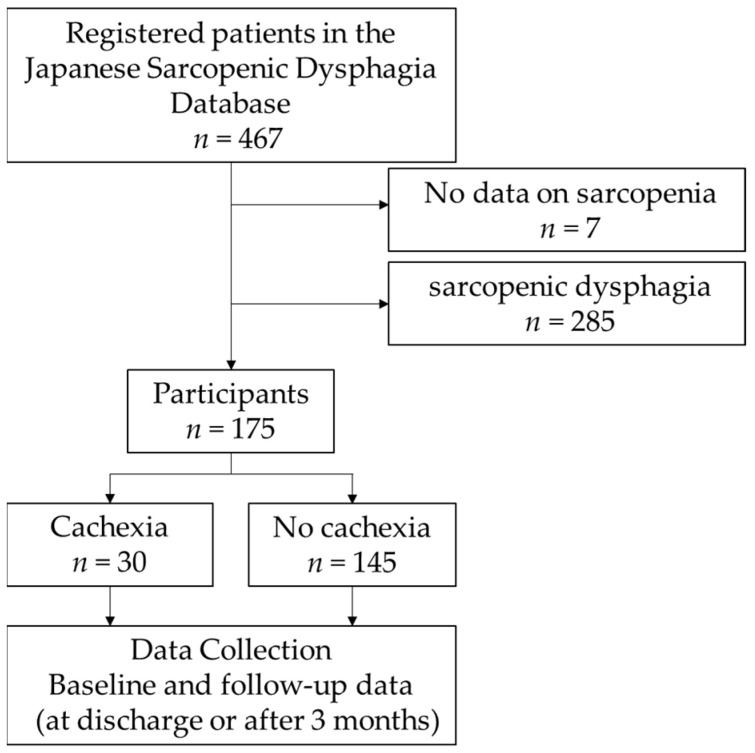
The flowchart of data collection for the participants. Out of the 467 patients enrolled in the database, 175 were included in the study and grouped based on the AWGC criteria for the presence or absence of cachexia. The data were collected at two time points: baseline and follow-up.

Of the 467 patients in the database, 7 without sarcopenia data and 285 with sarcopenic dysphagia were excluded, leaving 175 patients who met the selection criteria (Figure 1).

Table 1 presents the characteristics of the patients at baseline and follow-up. Cachexia was present in 30 of 175 patients (17%). Prognosis at follow-up included seven patient deaths (4%), a median (IQR) FILS score of 8 (7, 8), and a median (IQR) BI of 50 (20, 85). For the other data, the mean age was 84 (±8) years, and 103 (59%) were men. The causative diseases of dysphagia were stroke (105; 60%), parkinsonism (15; 9%), dementia (12; 7%), cancer (8; 5%), and others (35; 20%). Patients had a prevalence of existing chronic diseases such as cancer (13; 7%), chronic heart failure (21; 12%), chronic renal failure (8; 5%), and chronic obstructive pulmonary disease (8; 5%). Of the 30 patients with cachexia, 4 did not have sarcopenia, and 11 did not have malnutrition. Additionally, cachexia patients were more commonly found in acute care hospitals (23; 77%).

The univariate analysis revealed that the cachexia group had a significantly higher risk of death (5/30; 17% vs. 2/145; 1%, *p* = 0.002). Median FILS (7 vs. 8, *p* = 0.585) and BI (35 vs. 50, *p* = 0.469) scores were not significantly different between patients with and without cachexia. The small number of deaths precluded conducting a multivariate analysis.

**Figure 2:**
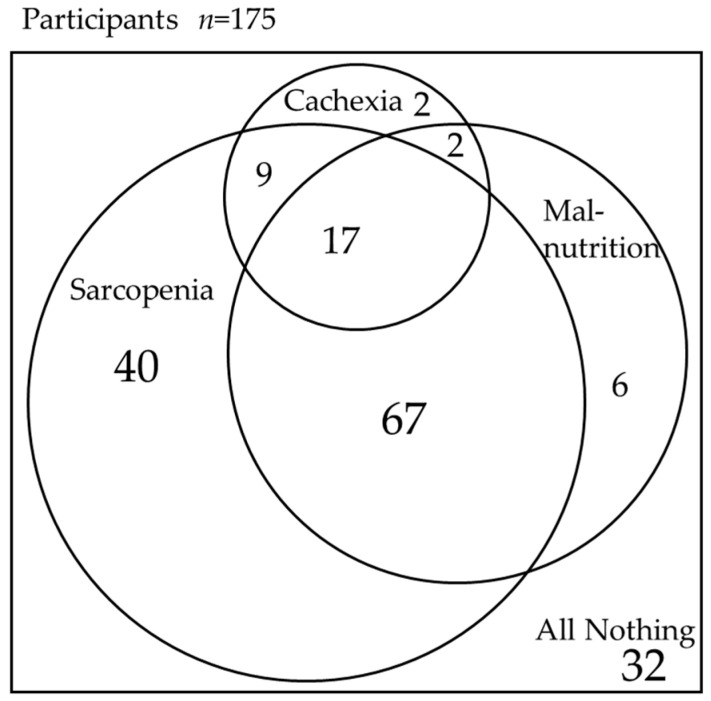
The relationship between cachexia, sarcopenia, and malnutrition among participants. The overlapping circles indicate the overlap of each factor, and the numbers represent the number of people with each factor. Cachexia (+), sarcopenia (+), and malnutrition (+): *n* = 17. Cachexia (+), sarcopenia (+), and malnutrition (−): *n* = 9. Cachexia (+), sarcopenia (−), and malnutrition (+): *n* = 2. Cachexia (+), sarcopenia (−), and malnutrition (−): *n* = 2. Cachexia (−), sarcopenia (+), and malnutrition (+): *n* = 67. Cachexia (−), sarcopenia (+), and malnutrition (−): *n* = 40. Cachexia (−), sarcopenia (−), and malnutrition (+): *n* = 6. Cachexia (−), sarcopenia (−), and malnutrition (−): *n* = 32.

Figure 2 shows the percentage of patients with and without cachexia, sarcopenia, and malnutrition. Of the 30 patients with cachexia, 2 patients (6%) had cachexia and neither sarcopenia nor malnutrition, 9 patients (30%) had cachexia and sarcopenia but no malnutrition, 2 patients (6%) had cachexia and malnutrition but no sarcopenia, and 17 patients (57%) had cachexia, sarcopenia, and malnutrition.

Table 2 shows the causative disease of admission and cause of death, in addition to the presence or absence of sarcopenia, malnutrition, and cachexia. All seven deceased patients had sarcopenia, and only those with systemic metastatic cancer did not have malnutrition. Of the five patients with cachexia who died, two (with systemic metastatic cancer and colorectal cancer) died of worsening cachexia, and three died of other causes, although the cause of one death was unknown. Four of the five deaths in the cachexia group were male.

## 4. Discussion

This study examined the prevalence and prognosis of cachexia as defined by the AWGC criteria in patients with non-sarcopenic dysphagia, revealing three key findings. First, cachexia was identified in 17% of these patients. Second, death was more frequent among patients with cachexia. Third, some patients with cachexia did not exhibit sarcopenia or malnutrition.

Cachexia was present in 17% of patients with non-sarcopenic dysphagia. The prevalence of cachexia ranged from 3.4% to 66.2% in non-cancer patients and from 6.2% to 93% in cancer patients, which is consistent with our findings [32]. We previously reported on 271 patients with sarcopenic dysphagia, with a mean age of 84 (±8) years, including 119 (44%) men, 97 (36%) with cachexia, and 160 (59%) with comorbidities attributable to cachexia (such as cancer, congestive heart failure, chronic obstructive pulmonary disease, and chronic renal failure) [21]. Compared to our previously reported sarcopenic dysphagia patients, the non-sarcopenic dysphagia patients in this study were, on average, seven years younger, had a 15% higher proportion of men, a 19% lower incidence of cachexia, and a 20% lower incidence of comorbidities related to cachexia. Men have a higher prevalence of cachexia and higher rates of weight loss and mortality [33]. The risk of cachexia increases with age [34]. In our study, although a more significant proportion of men were present, a younger age and fewer comorbidities seemed to contribute to the lower rate of cachexia.

The death rate for cachexic patients with non-sarcopenic dysphagia was 17%, significantly higher than the 1% death rate for non-cachexic patients. Four of the five deaths in the cachexia group were male. One factor associated with the prognosis of cancer cachexia is gender, with males having a worse prognosis [33,35,36]. Gender differences in muscle composition and metabolism are relevant. Men typically have a higher proportion of type 2 muscle fibers, which are more susceptible to cancer-induced muscle loss. In contrast, women have higher estrogen levels, a crucial hormone for protecting muscles, promoting muscle regeneration, and safeguarding mitochondrial function [33,37,38]. However, few studies have explored sex differences in cachexia outside of cancer patients, and in our study, the association between cachexia, sex, and mortality in non-cancer patients remains unclear. BMI was also significantly lower, and the rate of weight loss at six months tended to be higher in our study. High rates of weight loss also contribute to mortality [39]. The prevalence of the AWGC criteria for cachexia in 1330 postoperative gastric cancer patients was 461 (34.7%), and cachexia was associated with overall survival [15]. In 364 advanced cancer patients receiving palliative care, the prevalence of cachexia was 277 (76%), and overall survival was shorter for those with cachexia [14]. Among 367 hemodialysis patients, 129 (35.1%) met the criteria for cachexia; however, cachexia was not associated with overall survival [17]. Caution should be exercised in cases of refractory cachexia with short survival, as the AWGC criteria for cachexia may not be a good prognostic predictor [40]. In patients with non-sarcopenic dysphagia, mortality may vary by sex, weight loss, and comorbidity.

Among patients with cachexia, 6% had neither sarcopenia nor malnutrition, while 57% had both conditions. Additionally, cachexia was associated with the cause of death in some patients. The high percentage of patients in acute care hospitals and with elevated CRP levels may indicate that acute illness leads to acute sarcopenia [10,41,42,43]. Complications arising from sarcopenia and malnutrition worsen the life expectancy and increase the functional disability in patients with cachexia [22,44]. In hospital-associated sarcopenia, which is sarcopenia caused by hospitalization, the prevention of iatrogenic sarcopenia is essential [45]. A multimodal approach combining exercise and nutrition helps treat sarcopenia and malnutrition [46,47]. However, some cancer and osteoporotic patients with sarcopenia, malnutrition, and cachexia may inadequately adhere to therapy [13,48,49]. Adherence is often hindered by cachexia-causing diseases, comorbidities, aging, polypharmacy, and psychological barriers [49,50]. Multidisciplinary interventions, including nutritional and exercise therapy, along with medication management and patient education on topics such as smoking cessation and alcohol abstinence, are crucial. Additionally, support for patient decision-making, continuous evaluation and monitoring, and efforts to enhance patient satisfaction can help improve adherence [49,51]. Early assessment for cachexia, sarcopenia, and malnutrition, along with interventions such as exercise, nutrition, psychology, and medication, may improve physical function, ADLs, and QOL [13,52,53,54,55,56].

Our study has several limitations. First, the number of deaths was too small (*n* = 7) to perform a multivariate analysis, so it is unclear whether cachexia affects mortality more than other factors. Second, the severity and duration of each disease are unknown from the database. The presence of unrecorded comorbidities may have increased the rate of cachexia. Third, the long-term effects of cachexia have not been addressed. Finally, we did not assess QOL, one of the clinical outcomes recommended by the AWGC. Future studies should consider QOL to increase the robustness of these findings.

## 5. Conclusions

Cachexia was present in 17% of patients with non-sarcopenic dysphagia. Death rates were 17% in the cachexia group and 1% in the non-cachexia group. Among patients with cachexia, 6% did not have whole-body sarcopenia or malnutrition, while 57% had both conditions. An early assessment of cachexia, sarcopenia, and malnutrition and interventions such as exercise and nutritional therapy may help in the prevention and treatment of these conditions.

## Figures and Tables

**Table 1:** The characteristics of the patients at baseline and follow-up.

	Total *n* = 175	Cachexia (+) *n* = 30	Cachexia (−) *n* = 145	*p*-Value
Age, years, mean ± SD *	77 ± 11	78 ± 9	77 ± 11	0.437 ^a^
Sex, *n* (%)				0.103 ^b^
Men	103 (59%)	22 (73%)	81 (56%)	
Women	72 (41%)	8 (27%)	64 (44%)	
Setting, *n* (%)				0.001 ^b^
Acute care hospitals	78 (45%)	23 (77%)	55 (38%)	
Rehabilitation hospitals	76 (43%)	4 (13%)	72 (50%)	
Others	21 (12%)	3 (10%)	18 (12%)	
Main causative diseases of dysphagia, *n* (%)				
Cerebral infarction	77 (44%)	9 (30%)	68 (47%)	0.113 ^b^
Cerebral hemorrhage	19 (11%)	2 (7%)	17 (12%)	0.536 ^b^
Subarachnoid hemorrhage	9 (5%)	0 (0%)	9 (6%)	0.361 ^b^
Parkinsonism	15 (9%)	5 (17%)	10 (7%)	0.142 ^b^
Dementia	12 (7%)	3 (10%)	9 (6%)	0.435 ^b^
Cancer	8 (5%)	7 (23%)	1 (1%)	<0.001 ^b^
Others	35 (20%)	4 (13%)	31 (21%)	0.453 ^b^
Comorbidities, *n* (%)				
Chronic heart failure	21 (12%)	9 (30%)	12 (8%)	<0.001 ^b^
Cancer	13 (7%)	15 (50%)	8 (6%)	<0.001 ^b^
Chronic renal failure	8 (5%)	3 (10%)	5 (3%)	0.139 ^b^
Chronic obstructive pulmonary disease	8 (5%)	4 (13%)	4 (3%)	0.030
Chronic respiratory failure	0 (0%)	0 (0%)	0 (0%)	-
Progressive worsening or uncontrolled chronic infections	0 (0%)	0 (0%)	0 (0%)	-
Chronic liver failure	0 (0%)	0 (0%)	0 (0%)	-
Rheumatoid arthritis and other collagen diseases	0 (0%)	0 (0%)	0 (0%)	-
Sarcopenia, *n* (%)	133 (76%)	26 (87%)	107 (74%)	0.133
Malnutrition, *n* (%)	92 (53%)	19 (63%)	73 (50%)	0.181
Body mass index, kg/m^2^, mean ± SD *	21.0 ± 4.0	18.9 ± 3.0	21.4 ± 4.0	<0.001 ^a^
Body weight change in 6 months, %, median (IQR **)	3.3 (−1.9, 12.7)	11.0 (3.3, 19.7)	1.5 (−3.5, 10.5)	0.074 ^c^
Handgrip strength, kg, mean ± SD *	16.5 ± 10.9	15.2 ± 10.2	16.8 ± 11.1	0.467 ^a^
C-reactive protein, mg/dL, median (IQR **)	0.5 (0.1, 1.9)	3.4 (0.8, 11.6)	0.3 (0.1, 1.0)	<0.001 ^c^
FILS *** initial, median (IQR **)	7 (1, 7)	2 (1, 7)	7 (1, 7)	0.160 ^c^
FILS follow-up, median (IQR **)	8 (7, 8)	7 (5.5, 8)	8 (7, 8)	0.585 ^c^
Barthel Index initial, median (IQR **)	25 (10, 50)	25 (5, 67.5)	30 (10, 50)	0.406 ^c^
Barthel Index follow-up, median (IQR **)	50 (20, 85)	35 (12.5, 75)	50 (20, 85)	0.469 ^c^
Death, *n* (%)	7 (4%)	5 (17%)	2 (1%)	0.002 ^b^

* SD: standard deviation, ** IQR: interquartile range, *** FILS: Food Intake Level Scale, a: *t*-test, and b: Chi-square, c: Mann-Whitney U test.

**Table 2:** The causative disease of admission, the cause of death, and sex, in addition to the presence or absence of sarcopenia, malnutrition, and cachexia.

No	Sex	Causative Disease of Admission	Cause of Death	Sarcopenia	Malnutrition	Cachexia
1	Woman	Urinary tract infection	Senility	+	+	+
2	Man	Gastric cancer	Systemic metastatic cancer	+	−	+
3	Man	Colorectal cancer	Colorectal cancer	+	+	+
4	Man	Urinary tract infection	Pyothorax	+	+	+
5	Man	Cardiogenic cerebral embolism	Unknown	+	+	+
6	Woman	Cerebral infarction	Stroke	+	+	−
7	Woman	Multiple system atrophy	Septic shock	+	+	−

## Data Availability

The datasets presented in this article are not readily available because the data are part of an ongoing study.
